# Multi-omics approaches to understand pathogenicity during potato early blight disease caused by *Alternaria solani*

**DOI:** 10.3389/fmicb.2024.1357579

**Published:** 2024-03-11

**Authors:** Qing Li, Yan Feng, Jianmei Li, Yang Hai, Liping Si, Chen Tan, Jing Peng, Zuo Hu, Zhou Li, Canhui Li, Dahai Hao, Wei Tang

**Affiliations:** ^1^Yunnan Key Laboratory of Potato Biology, Yunnan Normal University, Kunming, China; ^2^School of Life Sciences, Yunnan Normal University, Kunming, China; ^3^School of Economics and Management, Yunnan Normal University, Kunming, China; ^4^Yunnan YinMore Modern Agriculture Co., Ltd., Kunming, China; ^5^Zhaotong Academy of Agricultural Sciences, Zhaotong, China

**Keywords:** potato early blight, *Alternaria solani*, *Alternaria alternata*, genome, transcriptome, proteome, pathogenicity

## Abstract

Potato early blight (PEB), a foliar disease of potato during the growing period, caused by *Alternaria* sp., is common in major potato-producing areas worldwide. Effective agents to control this disease or completely resistant potato varieties are absent. Large-scale use of fungicides is limited due to possibility of increase in pathogen resistance and the requirements of ecological agriculture. In this study, we focused on the composition and infection characteristics of early blight pathogens in Yunnan Province and screened candidate pathogenesis-related pathways and genes. We isolated 85 strains of *Alternaria* sp. fungi from typical early blight spots in three potato-growing regions in Yunnan Province from 2018 to 2022, and identified 35 strains of *Alternaria solani* and 50 strains of *Alternaria alternata* by morphological characterization and ITS sequence comparison, which were identified as the main and conditional pathogens causing early blight in potato, respectively. Scanning electron microscope analysis confirmed only *A. solani* producing appressorium at 4 h after inoculation successfully infected the leaf cells. Via genome assembly and annotation, combine transcriptome and proteomic analysis, the following pathogenicity-related unit, transcription factors and metabolic pathway were identified: (1) cell wall-degrading enzymes, such as pectinase, keratinase, and cellulase; (2) genes and pathways related to conidia germination and pathogenicity, such as ubiquitination and peroxisomes; and (3) transcription factors, such as *Zn-clus*, *C2H2*, *bZIP*, and *bHLH*. These elements were responsible for PEB epidemic in Yunnan.

## 1 Introduction

Globally, potatoes constitute the fourth largest staple crop being grown extensively in many regions. However, the production of potatoes is affected by several pathogens, such as bacteria, viruses, oomycetes, and fungi (Loebenstein and Gaba, 2012; Okiro et al., 2022; Osdaghi et al., 2022; Tiwari et al., 2022; Angmo et al., 2023). Potato early blight (PEB), caused by genus of *Alternaria*, is a fungus disease attacked both leaf and tubers during the growth season. In the early infection stages, older leaves at the bottom of the plant developed irregular or regular small brownish-black spots. In the later stages, leaves displayed deep brown or black spots with necrotic centers, sometimes resembling concentric circles (Chaudhary et al., 2021). Subsequently, the upper leaves and stems of the plant got infected. PEB on tubers were appeared brown skin protrusions with circular to irregular depressions; these marginal depressions eventually got soft and rot. The PEB disease could occur and spread in most weather conditions. The conidia of the dropped leaves or residual branches could lead to cyclic infections, exacerbating or advancing the onset of the disease. Reportedly, PEB resulted in a 40%–50% loss in potato yield worldwide (Abuley and Nielsen, 2017; Chaudhary et al., 2021).

Fungus of the genus *Alternaria* typically produced colored chain-grid-like conidia (brown to yellow-brown or dark brown). These conidia could have horizontal, vertical, or diagonal separations; further, they had a typical long beak. Different species of *Alternaria* can be further classified into large and small conidia-producing species. Large-spored species, such as *Alternaria solani* (Chaudhary et al., 2021), *Alternaria grandis* (Rodrigues et al., 2010; Da Silva et al., 2021), *Alternaria protenta* (Ivanović et al., 2022), and *Alternaria linariae* (Ayad et al., 2018), were considered the main pathogens causing early blight and leaf spot diseases in *Solanaceous* plants. Small-spored species, such as *Alternaria interrupta* (Taheri et al., 2009), *Alternaria infectoria* (Kokaeva et al., 2018), *A. alternata* (Zheng et al., 2015), and *Alternaria tenuissima* (Zheng et al., 2015; Taheri, 2019), were frequently isolated from symptomatic tissues also.

Pathogenic microorganisms infiltrated plant cells via stomata or wounds could trigger plant’s pattern recognition receptors (PRRs) that recognized pathogen-associated molecular patterns (PAMPs) in a defense mechanism called PAMP-triggered immunity (PTI) that blocked further colonization of pathogens. However, toxins released by pathogenic microorganisms could interfere with PTI, initiating a pathogenesis response called effector triggered susceptibility (ETS). In response, plants produced nucleotide-binding and leucine-rich repeat domain (NB-LRR) proteins that specifically recognized and generated a more potent effector-triggered immunity (ETI), effectively stopping the growth and reproduction of pathogenic microorganisms. Pathogenic microorganisms had even evolved strategies, such as hiding or differentiating recognized effector molecules, to evade ETI. Moreover, pathogens acquire additional effector molecules to suppress ETI (Jones and Dangl, 2006; Zhang S. et al., 2022). Genome mapping could identify specific structures of effectors and virulence genes (Zhang et al., 2018), and analyzing differences in transcriptomes over time could improve our understanding of pathogenic mechanisms. Using high-resolution mass spectrometry-based quantitative proteomics had also been widely used to understand plant-pathogen interactions (Kim et al., 2021). Furthermore, plant pathogens usually colonized specific cell types and tissues of the plant, and scanning electron microscopy could be used to better understand the interactions between plant pathogens and specific cells, enabling elucidation of pathogen lifestyles and virulence mechanisms (Caldwell and Iyer-Pascuzzi, 2019).

In China, Yunnan Province is one of the primary potato production area. In recent years, there had been an increase in the incidence of early blight disease, with a complex structure of the pathogenic population. In order to clarify the composition, infection characteristics and pathogenicity of PEB pathogens in Yunnan Province in recently years, in this study, we collected (2018–2022) infected potato leaves from major cultivars in Yunnan and identified the types of strains isolated based on morphology and ITS sequencing. The pathogenicity of the isolates was then verified and the main pathogens causing PEB were identified. We further explored the genome assemble to pathogenic mechanism and genes involved analysis during PEB infection process using scanning electron microscopy and genomic, transcriptomic, and proteomic analyses.

## 2 Materials and methods

### 2.1 Isolation and identification of strains

We collected (2018–2022) leaves from potato plants with the early blight disease from three regions of Yunnan Province (Heqing, Jianchuan, Eryuan district in Dali, Ninglang country in Lijiang, and Malong district in Qujing) (Supplementary Table 1). Leaves were thoroughly rinsed with sterile water and air dried. The leaves were then disinfected with 0.3% sodium hypochlorite solution for 1 min and surface sterilized using 75% alcohol for another minute. Leaves were repeatedly rinsed three times with sterile water, followed by air drying. Subsequently, the leaves were placed on PDA medium and incubated at 26°C and observed for asexual conidia after 7 days under a microscope (40× magnification). The conidia were then isolated and purified, and this process was repeated thrice to obtain pure cultures. Strains were identified based on their morphological characteristics and ITS sequencing.

### 2.2 Pathogenicity test

Conidial suspensions were prepared by incubating the isolates on PDA solid medium at 25°C for 7 days. Conidia were then scrapped off after adding 1 ml of sterile water. This suspension (1 μl) was used to measure the concentration of the conidia. This process was repeated three times, and the average concentration of the conidia was determined to 3 × 10^4^ conidia/ml. The conidia suspension was stored at 4°C for 1 h to promote germination. We took 20 μl of each isolate and inoculated it on Cooperation 88 (C88) leaves, each strain inoculated three leaves and the experiment was repeated three times. Imaging of leaves 168 h after inoculation and calculation of lesions incidence, the calculation formula is as follows (Zhang D. et al., 2022). Disease severity measured on a scale of 0–5: (1) level 0: no symptoms, resistant; (2) level 1: 1%–5% of spots on the leaves, moderately resistant; (3) level 2: 6%–20% of spots on the leave, moderately resistant; (4) level 3: 21%–40% of spots on the leaves, moderately susceptible; (5) level 4: 41%–60% of spots on the leaves, moderately susceptible; and (6) level 5: more than 61% of spots on the leaves, susceptible (Satti et al., 2021).

Diseasedleafrate(%)

=Numberofdiseasedleaves/Totalleavesinoculated)×100%.

Most isolates were from the same leaf, and we set up the following experiments to further investigate the early blight infection pattern. The most virulent of the isolates, No. TA-0410, was selected, and another strain, TB-1129, was isolated from the same spot as TA-0410. The healthy leaves of potato varieties C88 and Desiree tubers 45 days after planting were used for inoculation with conidia. The following six treatment groups were set up: (1) in group A, inoculation was conducted using 10 μl of sterile water at two symmetrical points, (2) in group B, 10 μl of TA-0410 conidia suspension was placed at two symmetrical points, (3) in group C, 10 μl of TB-1129 conidia suspension was placed at two symmetrical points, (4) in group D, 5 μl of TA-0410 conidia suspension was placed, and after 12 h, 5 μl of TB-1129 conidia suspension was placed at the same inoculation point, (5) in group E, 5 μl of TB-1129 conidia suspension was inoculated, and after 12 h, 5 μl of TA-0410 conidia suspension was added at the same inoculation point, and (6) in group F, a mixture (10 μl) of equal volumes of TA-0410 and TB-1129 conidia suspensions was placed at two symmetrical points. Leaves of all groups were then placed in a light incubator at 26°C, with 1,500 lx (16 h of daylight and 8 h of darkness) and 85% humidity and were observed (after turning the leaves face up) for disease symptoms after 24 h of inoculation (12 h after the second inoculation in groups D and E). A total of 10 leaves were inoculated in each group, and the entire experiment was conducted three times. Leaves were imaged and lesions incidence were calculated.

### 2.3 Infection characteristics of PEB

The edges of the colonies of TA-0410 and TB-1129 were collected as 5 mm pieces and inoculated onto the PDA medium. Subsequently, the conidia production was measured after incubation at 25°C for 5 days. In this method, we added 5 ml of sterile water to the Petri dish and used a scalpel to scrape a colony several times to scrape off the conidia produced by the strain. This allowed us to make a well-mixed conidia suspension. Next, TA-0410 and TB-1129 strains were incubated at 25°C for 7 days, followed by the addition of 1 ml of PDA medium and scrapping of the conidia using a coating rod. The concentration of conidia suspension was determined by drawing 1 μl of the conidia suspension. The average final concentration of conidia was found to be 3 × 10^4^ conidia/ml. We then measured the conidia germination rate at 4, 8, 12, and 24 h by incubating 20 μl of the conidia suspension individually.

We infected C88 leaves using the conidia suspensions of TA-0410 and TB-1129 strains and collected samples (in triplicates) at 4, 8, 12, and 24 h post-inoculation. Collected samples were fixed using 3% glutaraldehyde, washed thrice using ultrapure water for 10 min, fixed again using 1% osmium tetroxide for 1 h, and washed again thrice with ultrapure water for 10 min. Samples were dehydrated gradually using a gradient of alcohol as follows: 30% – 50% – 70% – 80% – 90% – 95% – 100% (with the 100% concentration applied three times); each dehydration step applied for 15 min. The samples are then adhered to a sample holder with conductive adhesive and coated in an ion sputter. Samples were images using a JSM-IT700HR scanning electron microscope (Japan Electron Optics Laboratory Ltd.). The germination of TA-0410 and TB-1129 conidia on C88 leaves was recorded at 4, 8, 12, and 24 h post-inoculation.

### 2.4 Genomic analysis

Genomic DNA was extracted from TA-0410 and TB-1129 (cultured on PDA medium and incubated for 7 days at 26°C) using an E.Z.N.A.^®^ Fungal DNA Kit (Omega Bio-Tek Inc., China). The concentration of the DNA was measured using a NanoDrop 1000 (NanoDrop Technologies Inc., USA), and purity was ensured by evaluating the ratio of absorbance values determined at 260 and 280 nm (260/280 nm). Whole genome sequencing was performed by Beijing Novogene Bioinformatics Technology Co., Ltd. using an Illumina NovaSeq 6000 PE150 and HiFi system, with a sequencing depth of 30× and 20×, respectively. The sequenced sequences are as follows: (1) ILLUMINA platform: P5: 5′-AATGATACGGCGACCACCGA-3′ and P7: 5′-CAAGCAGAA GACGGCATACGAGAT-3′; (2) PacBio platform: 5′-adapter: AA GTCGGATCGTAGCCATGTCGTTCTGTGAGCCAAGGAGTTG and 3′-adapter: AAGTCGGAGGCCAAGCGGTCTTAGGAAGA CAA. Raw reads were quality-checked and filtered to remove low-quality reads, reads containing sequencing adapters, and reads with N bases. The resulting HiFi clean reads were subsequently assembled *de novo* using the CLC Genomics Workbench 20.0.3 (QIAGEN CLC Genomics Workbench, Denmark) software, with assembly parameters set at word size = 17 and bubble size = 50. Gaps were polished by clean reads from Illumina sequencing in CLC long read support module. The distribution of genomic GC content was also determined. Assembled genomes were evaluated using the BUSCO’s Fungi and Ascomycota databases. CDS were predicted and *de novo* annotated using OmicsBox 3.0. All putative proteins of TA-0410 and TB-1129 were searched against entries in the CAZy database by using CAZymes Analysis Toolkit using the Carbohydrate Active Enzymes (CAZy) database.^1^ The parameter values were in default on the website. All identified proteins were then manually retrieved.

### 2.5 Transcriptome sequencing

The healthy potato variety (C88) was inoculated with TA-0410. The control group (inoculation using sterile water) was also setup. Samples were collected from the infected and control groups at initial (24 h), mid (96 h), and late (168 h) stages of infection. Although TB-1129 lacks the ability to directly infect C88, transcriptome analysis was conducted to understand transcriptional basis of disease aggravation in situations with plant wounds. Samples were collected from the infected and control groups at the 96 h of infection. All collected samples were sent to Wuhan Kangce Technology, China for library construction and Illumina sequencing. Sequencing data were first evaluated for quality using the fastp software (version 0.23.0), and the clean data were aligned with the reference genome using STAR (version 2.7.6a). FeatureCounts (version 1.5.1) was used to calculate unique reads aligned to the reference genome and genes and to determine gene expression levels. Differentially expressed genes were analyzed using EdgeR (version 3.28.1), with a standard of absolute logFC value >1 and *P*-value < 0.05. Functional annotations were conducted using databases, Uniprot, NR, Refseq, Pfam, and Interpro. Kobas software (version 2.1.1) was used to annotate the biological processes, cellular components, and molecular function classifications involved in the transcriptome at different times in the gene ontology (GO) database. Metabolic pathway-related information was obtained using the Kyoto encyclopedia of genes and genomes (KEGG) database.

### 2.6 Proteomic analysis

The healthy potato variety (C88) was infiltrated with TA-0410. As control, the healthy potatoes were infiltrated with sterile water. After 24 h, samples were collected from infection spots and immediately placed in liquid nitrogen. Frozen samples were transported to Baimaike, Beijing, China for protein extraction, quality check, and enzymatic desalting of protein metabolomes using the Q Exactive HF-X mass spectrometer equipped with a Nanospray Flex™ (NSI) ion source. The ion spray voltage and ion transfer tube temperature were set at 2.4 kV and 275°C, respectively. The mass spectrometer used a data-dependent acquisition mode, with a full scan range of 350–1,550 m/z. The primary mass spectrometry resolution, AGC, and maximum injection time were set at 120,000 (200 m/z), 3 × 10^6^, and 80 ms, respectively. The top 40 parent ions were selected (based on intensity) from the full scans and fragmented using the high-energy collision dissociation (HCD) method for secondary mass spectrometry detection, with the following settings: resolution, 15,000 (200 m/z); AGC, 5 × 10^4^; the maximum injection time, 45 ms; and peptide fragmentation collision energy, 27%. Raw data were processed and used for quantitative expression analysis. The area of each protein peak in each sample was divided by the total peak area of all proteins in that sample to obtain protein expression levels. Subsequently, a principal component analysis was also conducted. The identified proteins were functionally annotated using GO, KEGG databases to understand the functional characteristics of different proteins.

### 2.7 Quantitative real-time PCR

Healthy potato variety (C88) was infected with TA-0410 for initial (24 h), mid (96 h), and late (168 h) stages, followed by RNA extraction using a plant RNA extraction kit (Omega, R6827-02, Guangzhou, China). cDNA was synthesized using reagents from Takara (TaKaRa, Beijing, China). Quantitative real-time PCR reaction was carried out using TB Green Premix Ex Taq II (TaKaRa, Beijing, China). The actin gene was used as the internal reference gene, and the 2^–ΔΔCt^ method was used to calculate relative gene expression with three biological replicates. Primers were designed online using NCBI Primer-BLAST with Actin as the internal reference gene, and the primer sequences are presented in Supplementary Table 6.

### 2.8 Statistical analysis

Three independent experiments were performed for each assay. Data were analyzed using SPSS 20.0 (SPSS Inc., Chicago, IL, United States). Significant differences were calculated to compare the results at the 0.05 level.

## 3 Results

### 3.1 Identification of candidate PEB pathogens

**FIGURE 1:**
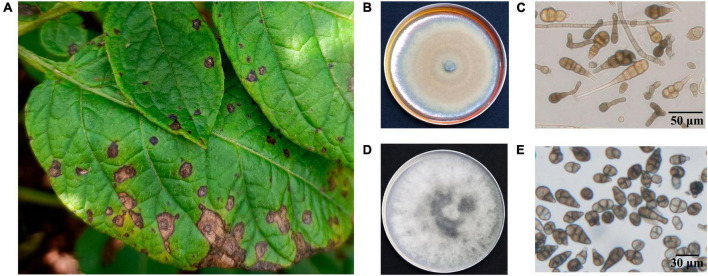
Characteristics of PEB disease and the morphology of causative pathogens. **(A)** Potato early blight symptoms on leaves. **(B)** Morphology of culture of *A. solani*. **(C)** Morphology of conidia of *A. solani*. **(D)** Morphology of culture of *A. alternata*. **(E)** Morphology of conidia of *A. alternata*.

In this study, we collected, isolated and purified 85 isolates for 35 *A. solani* and 50 *Alternaria alternata* and three other fungi from typical PEB leaves on different regions of Yunnan (Figure 1A and Supplementary Table 1). Morphology test showed mycelia of *A. solani* turned yellow-brown after 7 days of cultivation on PDA medium (Figure 1B). The conidia displayed an inverted club or grenade shape of color ranging from light yellow to brown. The conidia were mostly solitary; measured 37.4–151.9 (±28.1) μm × 4.3–22.9 (±4.1) μm; and had longitudinal (0–4, perpendicular to the beak), transverse (3–11, parallel to the beak), and oblique constricted (0–4) septa. The septas were light-colored and semi-transparent. The conidia contained 4–12 compartments, which were formed by septal division. The tails had a light-brown long [2.9–38.8 (±7.6)] μm beak often equaling to or longer than the conidia body. These morphological features were consistent to those reported for large-conidia species of *A. solani* (Zheng et al., 2015; Figure 1C). After 7 days of cultivation, the mycelia of *A. alternata* displayed the following features: gray-brown color (Figure 1D), cylindrical or grenade shape, usually solitary, with dimensions ranging from 18.6–42.6 (±9.3) μm × 6.1–15.3 (±2.3) μm, longitudinal (up to two) and transverse (one to six) constricted gray brown septa, two to seven compartments, with the tail having a light brown short beak, and beak length of 3.4–16.2 (±4.3) μm, which was usually shorter than the body of the conidia. These features were also consistent to those reported for *A. alternata* (Zheng et al., 2015; Figure 1E). The ITS sequencing results of representative strains *A. solani* TA-0410 and *A. alternata* TB-1129 had been uploaded to the NCBI database, with accession numbers OR486271 and OR485642, respectively.

### 3.2 The virulence of *A. solani* TA-0410 and *A. alternata* TB-1129

**FIGURE 2:**
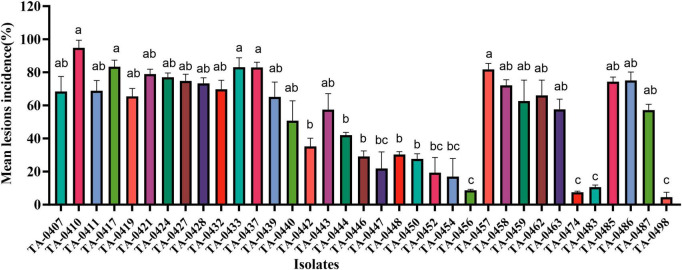
Results of virulence analysis of isolated strains. Different lowercase letters indicate significant difference at the *P* < 0.05 level.

We inoculated leaves of the PEB-resistant variety C88 with 85 isolates of *Alternaria* sp. statistical analyses of disease incidence and disease severity revealed that the most virulent of the 35 *A. solani* isolates was TA-0410, whereas none of the 50 *A. alternata* isolates could infect potato leaves (Figure 2 and Supplementary Table 2).

**FIGURE 3:**
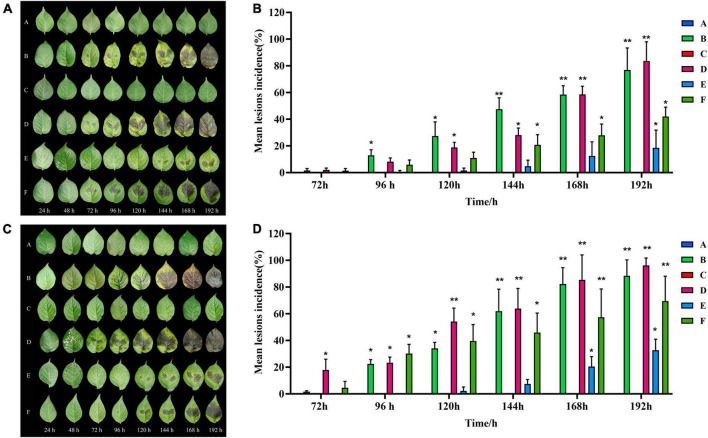
Pathogenicity test results and lesions incidence. **(A)** Pathogenic symptoms observed in the potato variety of C88. **(B)** Lesions incidence of potato variety C88 after different treatments. **(C)** Pathogenic symptoms in the potato variety of Desiree. **(D)** Lesions incidence of potato variety Desiree after different treatments. One-way ANOVA; *p < 0.05; **p < 0.01.

When infected with isolated strains, the leaves of healthy C88 plants started to display black-brown or brownish lesions within 72 h. These lesions were more advanced and severe in group D (inoculated with both TA-0410 and TB-1129). The severity of symptoms was then followed by group B (inoculated with TA-0410) and group F (inoculated with a spore mixture of TA-0410 and TB-1129). Group C, which was inoculated solely with TB-1129, did not display any symptoms (Figure 3A). In case of Desiree, visible lesions were observed in group B after 48 h; lesions continued to expand until 144 h, and at this time, rotting of leaves was started. In contrast, group C did not display any lesion even after 192 h of inoculation. Group D showed lesions 24 h post-inoculation, with the leaves starting to rot after 144 h. Group E displayed lesions 120 h after inoculation, but the expansion of these lesions was markedly slower compared with than in groups 2, 3, and 6. Group F displayed lesions 48 h after inoculation. In this group, the lesion area expanded rapidly after 168 h. Notably, the control group did not demonstrate any sign of disease until day 8 after inoculation. Desiree leaf inoculation results were consistent with C88 trends and were more severe than C88 (Figure 3). TA-0410 was more pathogenic than TB-1129 by calculating the lesions incidence (Figure 3). These results revealed that TA-0410 was the main pathogen causing the PEB disease. Furthermore, although TB-1129 could not directly cause the PEB disease, the pathogen could exacerbate early blight disease in potatoes infected by TA-0410.

### 3.3 Cellular impact of *A. solani* TA-0410 and *A. alternata* TB-1129 on potato leaves

**FIGURE 4:**
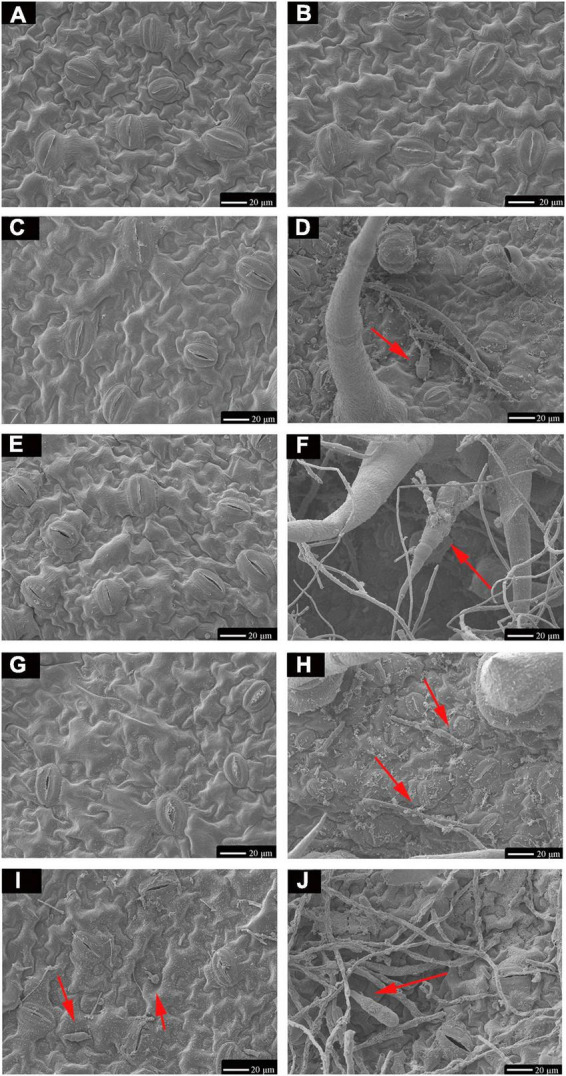
Scanning electron microscopy images (600×) of C88 leaf cells infected with TA-0410 and TB-1129. **(A)** Healthy cells not infiltrated by TB-1129. **(B)** Healthy cells not infiltrated by TA-0410. **(C)** TB-1129-infected C88 leaf cells observed after 4 h. **(D)** TA-0410-infected C88 leaf cells observed after 4 h, arrows point to conidia. **(E)** TB-1129-infected C88 leaf cells observed after 8 h. **(F)** TA-0410-infected C88 leaf cells observed after 8 h, the arrow pointed to conidia. **(G)** TB-1129-infected C88 leaf cells observed after 12 h. **(H)** TA-0410-infected C88 leaf cells observed after 12 h, arrows pointing to mycelium. **(I)** TB-1129-infected C88 leaf cells observed after 24 h, arrows point to conidia. **(J)** TA-0410-infected C88 leaf cells observed after 24 h, arrows point to conidia.

TA-0410 was identified as the main pathogen causing the PEB disease. We also found that TB-1129 could not directly cause the PEB disease; however, the pathogen could exacerbate early blight disease in potatoes infected by TA-0410. In the morphological studies using the scanning electron microscopy, healthy potato leaves demonstrated clearly visible structures (Figures 4A, B). In leaves infected with TB-1129, no significant structural changes were observed till 12 h, but at 24 h, a small amount of conidia germination was observed (Figures 4C, E, G, I). On the other hand, cells of TA-0410 infected leaves showed conidia germination as early as 4 h after inoculation, with hyphae spreading throughout the entire leaf cell (Figures 4D, F–H). In the infection stage, TA-0410 conidia and mycelium expanded in different directions and produced attachment cells, which was conducive to the invasion of the internal tissue structure. During the infection of potato leaf cells by TA-0410, the appressoria first secreted mucilage to enable them to adhere firmly to the host surface, which provided the impetus for the pathogen to penetrate the outer epidermis and cell wall of the host (Figure 4F).

**FIGURE 5:**
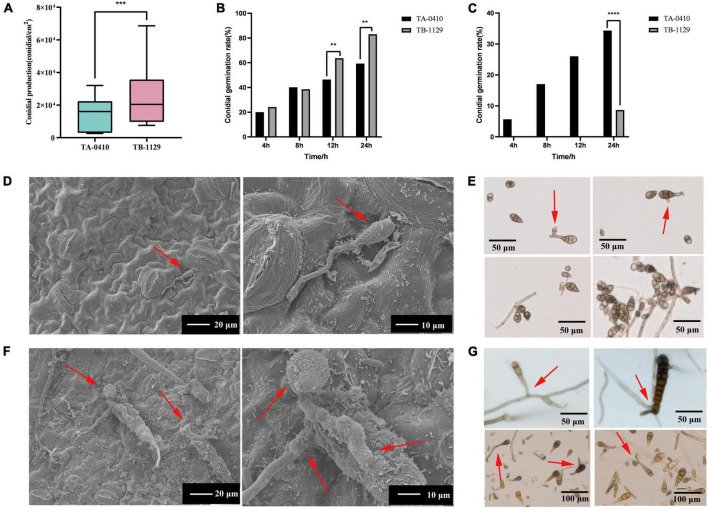
Conidia counts and germination morphology of leaves infected with TA-0410 and TB-1129. **(A)** TA-0410 and TB-1129 conidia production. **(B)** TA-0410 and TB-1129 conidia germination rate in PDA liquid medium. **(C)** TA-0410 and TB-1129 conidia germination rate after infection of C88 leaf cells. **(D)** Scanning electron micrographs of TB-1129 conidia 24 h after germination, arrow pointed to conidia. **(E)** Morphology of TB-1129 conidia, arrows pointed to areas of conidial germination. **(F)** Scanning electron micrograph of TA-0410 conidia 4 h after germination, arrows pointed to conidia and appressoria. **(G)** Morphology of TA-0410 conidia, arrows point to areas of conidial germination. One-way ANOVA; **p < 0.01; ***p < 0.001; ****p < 0.0001.

We next counted the conidia production in leaves infected with TA-0410 and TB-1129 under the same experimental conditions and found that the conidia production in TB-1129-infected leaves was significantly higher than that in TA-0410-infected leaves (Figure 5A). Furthermore, the conidia germination rate in TB-1129-infected leaves was also higher than that in TA-0410-infected leaves (Figure 5B). However, after inoculating the leaves with the same amount of conidia, conidia in leaves infected with TA-0410 and TB-1129 germinated at 4 and 24 h, respectively. Hence, the conidial germination rate of TA-0410 was higher than that of TB-1129 (Figure 5C). Observation by scanning electron microscopy revealed that TB-1129 germinated slowly, with spores germinating only after 24 h. Conidia produced attachment cells secreting a small amount of material, and conidia germinated irregularly, as shown by the arrows (Figures 5D, E). TA-0410 conidia germinated quickly and irregularly (Figures 5F, G), producing a large number of mycelium expanding and spreading in the C88 leaf cells, and the conidia produced appressoria secreting a large number of substances adsorbed to the C88 leaf cells. It was hypothesized that these substances helped TA-0410 to colonize on the one hand, and attacked the leaf cells on the other hand, contributing to leaf cell lesions.

### 3.4 Genome assembly and annotation

The assembled genome size of TA-0410 was found to be 33,130,331 bp, with 26 contigs (contig N_50_ was 2,688,818 bp) and a GC content of 52%. The assembled genome size of TB-1129 was 33,309,411 bp, with 92 contigs (scaffold N_50_ was 2,338,721 bp) and a GC content of 51.10%. When assembled genomes were aligned with the fungi and Ascomycota databases in BUSCO, a match rates of 98.8% and 97.7% (for TA-0410) and 98.8% and 97.2% (for TB-1129) were obtained. Genome sequencing data of TA-0410 and TB-1129 have been uploaded to the NCBI database, with accession numbers JAMBQH000000000 and JAHYXJ000000000, respectively. TA-0410 genome annotated 10,184 genes, whereas TB-1129 genome annotated 15,400 genes. Although the genetic composition of both strains were mostly similar, a few unique genes were identified in TA-0410. These uniques coded the following important proteins: (1) aegerolysin, which plays a role in conidia formation (Butala et al., 2017), and the fasciclin domain was crucial for conidia formation and pathogenicity (Seifert, 2018), (2) D-arabinono-1,4-lactone oxidase, which was located in mitochondria and was vital for conidia germination, appressorium formation, and pathogenicity in rice blast fungus (Wu et al., 2022), and (3) hydrophobins, which were closely related to conidia formation and pathogenicity (Bayry et al., 2012). It is hypothesized that most unique genes identified in the genome of TA-0410 were involved in conidia germination and virulence, whereas a few genes were involved in substance transport (Supplementary Table 3). Existence of these genes in the genome of TA-0410 might explain the ability of this strain to produce a large number of conidia and directly to infect potato leaf cells.

### 3.5 Analysis of cell wall-degrading enzymes of TA-0410

We identified 68 cell wall-degrading enzymes, such as pectinase, keratinase, and cellulase, in TA-0410. These enzymes are crucial for the ability of pathogens to invade and colonize plants cells and subsequently spread across host cells. TA-0410 was found to generate a series of enzymes capable of breaking down the cuticle and cell wall, enabling it to overcome the barrier of potato leaf cell walls and invade leaf cells. Hence, these enzymes were also closely related to virulence (Table 1).

**TABLE 1:** Cell wall-degrading enzymes of TA-0410.

CWDEs	Description (numbers)	PFAMs
Pectinase	Polysaccharide lyase family 3 protein (5)	Pectate_lyase
	Polysaccharide lyase family 1 protein (7)	Pec_lyase_C
	Pectinesterase (1)	Pectinesterase
	Pectin lyase (5)	Pectate_lyase
	Involved in maceration and soft-rotting of plant tissue (2)	Pectinesterase
	Glycoside hydrolase family 55 protein (4)	Pectate_lyase_3
	Glycoside hydrolase family 28 protein (1)	Pectinesterase
	Carbohydrate esterase family 8 protein (1)	Pectinesterase
	Amb_all (1)	CBM_1, Pec_lyase_C
Cutinase	Cutinase (2)	Cutinase
	Catalyzes the hydrolysis of cutin, a polyester that forms the structure of plant cuticle (5)	Cutinase
	Carbohydrate esterase family 5 protein (5)	Cutinase
	PE-PPE domain (1)	CBM_1, cutinase
Cellulase	Belongs to the glycosyl hydrolase 9 (cellulase E) family (1)	Glyco_hydro_9
	Belongs to the glycosyl hydrolase 7 (cellulase C) family (1)	Glyco_hydro_7
	Belongs to the glycosyl hydrolase 12 (cellulase H) family (4)	Glyco_hydro_12
	Belongs to the glycosyl hydrolase 11 (cellulase G) family (3)	Glyco_hydro_11
	Glycoside hydrolase family 5 protein (2)	Cellulase
	Glucan endo-1,6-beta-glucosidase activity (1)	Cellulase
	Cellulase (glycosyl hydrolase family 5) (2)	Cellulase
	Belongs to the glycosyl hydrolase 5 (cellulase A) family (13)	Cellulase
	Belongs to the glycosyl hydrolase 7 (cellulase C) family (2)	CBM_1, Glyco_hydro_7

### 3.6 Carbohydrate-active enzymes

**FIGURE 6:**
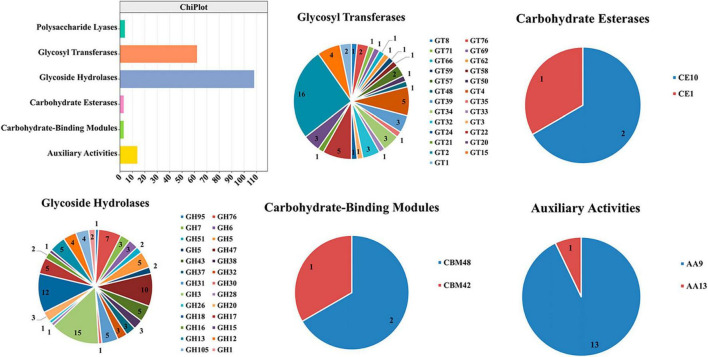
Carbohydrate-active enzymes were found in TA-0410.

The following were the five major categories of carbohydrate-active enzymes: glycoside hydrolases (GHs), glycosyl transferases (GTs), polysaccharide lyases (PLs), carbohydrate esterases (CEs), and auxiliary activities (AAs). Carbohydrate-binding modules (CBMs) also constitute a category of this type of enzymes. TA-0410 was identified to contain 108 GHs, 62 GTs, 14 AAs, 4 PLs, and 3 CBMs. CEs, GHs, and PLs were often referred to as cell wall-degrading enzymes. TA-0410 was found to harbor 108 GHs, 4 PLs, and 3 CEs. The localized degradation of the cell wall is essential for TA-0410 to enter potato leaf cells and spread to leaf tissues (Figure 6). We analyzed and compared the CAZymes of the two strains and found that TA-0410 contained more GH28 and GH43, etc., which are directly involved in cell wall degradation of the host plant, than TB-1129, which also facilitated the infection of potato (Zhao et al., 2013; Roy et al., 2023; Supplementary Table 4).

### 3.7 Transcriptomics analysis

**FIGURE 7:**
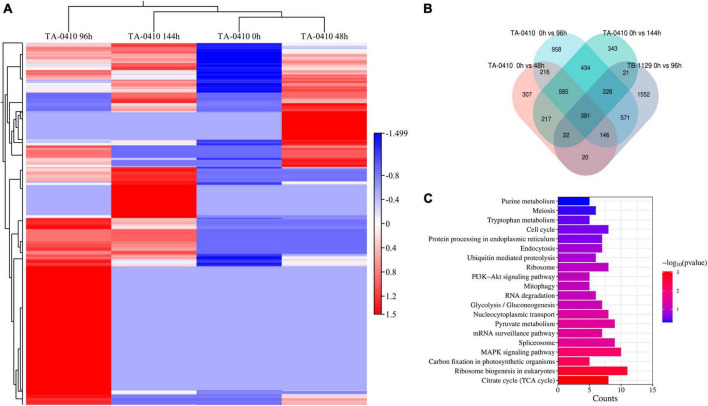
Transcriptomics analysis. **(A)** Heat map of differentially expressed genes at series infection times in TA-0410-infected leaf cells. **(B)** Wayne diagram of differentially expressed genes at series infection times in TA-0410 and TB-1129-infected cells. **(C)** KEGG enrichment of differentially expressed genes shared by TA-0410 and TB-1129-infected cells.

*Alternaria solani* TA-0410-infected C88 leaf cells had a total of 1,948, 3,608, and 2,295 differentially expressed genes at 48, 96, and 144 h, respectively (Figure 7A). Genes encoding *bZIP* transcription factor, ribosomal protein L9, AN1-like zinc finger, ClpA ClpB family, malate synthase family, and aldehyde dehydrogenase family proteins were found to be expressed increasingly at all three time points. *A. alternata* TB-1129-infected C88 leaf cells had a total of 3,027 differentially expressed genes at 96 h. Furthermore, 307, 958, and 343 genes were unique to TA-0410-infected C88 leaf cells at 48, 96, and 144 h, respectively. TB-1129-infected C88 leaf cells had 1,552 genes at 96 h (Figure 7B). We found that 391 genes were common between TA-0410 and TB-1129-infected cells; main enriched genes were related to ribosome biogenesis in eukaryotes (ko03008), MAPK signaling pathway (ko03008), signaling pathway (ko04011), spliceosome (ko03040), pyruvate metabolism (ko00620), TCA cycle (ko00020), nucleocytoplasmic transport (ko03013), ribosome (ko03010), and other basic biosynthetic pathways (Figure 7C).

**FIGURE 8:**
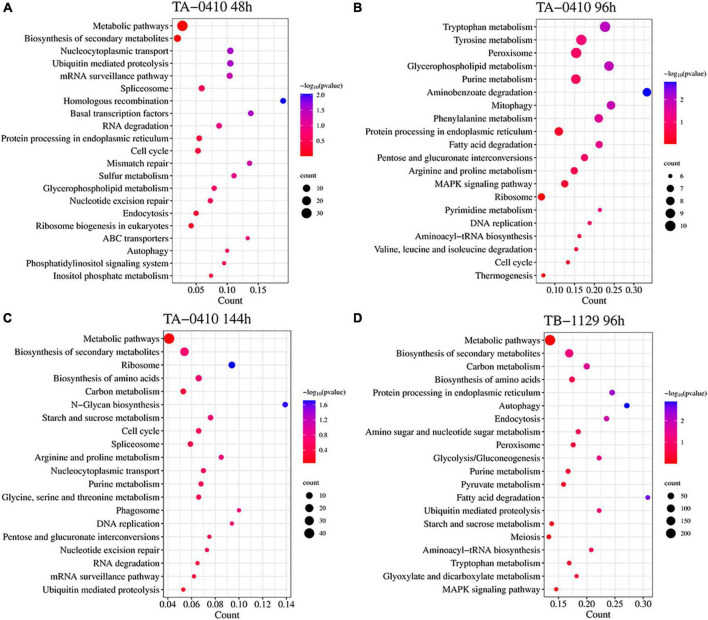
Transcriptomics and the KEGG pathway enrichment analysis of TA-0410 and TB-1129 infected C88 leaf cells. **(A)** TA-0410 at 48 h. **(B)** TA-0410 at 96 h. **(C)** TA-0410 at 144 h. **(D)** TB-1129 at 96 h.

In TA-0410-infected C88 leaf cells, GO functional enrichment analysis of unique, upregulated, differentially expressed genes at 48 h demonstrated the Biological Process as mainly enriched in cellular process (GO:0009987), metabolic process (GO:0008152), and single-organism process (GO:0044699); the Cellular Component was mainly enriched in cell (GO:0005623), cell part (GO:0044699 and 0044464), and intracellular (GO:0005622); and the Molecular Function was mainly enriched in catalytic activity (GO:0003824), binding (GO:0005488), and heterocyclic compound binding (GO:1901363) (Table 2). The KEGG pathway analysis was mainly enriched in metabolic pathways, biosynthesis of secondary metabolites, nucleocytoplasmic transport, and ubiquitin-mediated proteolysis (Figure 8A). Secondary metabolites usually confer ecological advantages to fungi and were essential for their ability to colonize and infect plants; however, these metabolites were not necessary for their survival. These secondary metabolites included substances secreted by conidia (as observed in scanning electron microscopy of TA-0410-infected C88 cells (Figure 4). These substances attached to C88 leaf cells to promote TA-0410 infection and colonization. In fungal pathogens, ubiquitin genes had been shown to play important roles in fungal development, stress resistance, and fungal virulence (Chen et al., 2018; Yang et al., 2020; Cao and Xue, 2021). Transcription factors (TFs) regulate different aspects of fungal development and virulence (Shelest, 2008; John et al., 2021). Further, sulfur metabolism is closely related to pathogenicity in fungi (Amich, 2022). Differentially expressed genes at 48 h of infection were mainly involved in the growth and development; at this time point, the expression of virulence-related genes started.

**TABLE 2:** Gene ontology enrichment analysis of differentially expressed genes found upregulated in TA-0410-infected cells at 48 h.

Terms	ID	Description	Gene ratio	*P*-value	Number
Biological process	GO:0009987	Cellular process	0.047	0.452801964	156
	GO:0008152	Metabolic process	0.046	0.599068655	130
	GO:0044699	Single-organism process	0.048	0.237689107	129
	GO:0044237	Cellular metabolic process	0.046	0.565111508	123
	GO:0071704	Organic substance metabolic process	0.045	0.739902352	119
	GO:0044763	Single-organism cellular process	0.048	0.304008475	118
	GO:0044238	Primary metabolic process	0.047	0.448420323	117
	GO:0006807	Nitrogen compound metabolic process	0.048	0.336506428	111
	GO:0043170	Macromolecule metabolic process	0.050	0.121123347	102
	GO:0044260	Cellular macromolecule metabolic process	0.049	0.247284986	95
	GO:0071840	Cellular component organization or biogenesis	0.050	0.169632474	87
	GO:0065007	Biological regulation	0.056	0.012990821	84
	GO:0016043	Cellular component organization	0.055	0.023175808	84
	GO:0034641	Cellular nitrogen compound metabolic process	0.046	0.58978566	79
	GO:0050789	Regulation of biological process	0.057	0.010084747	75
	GO:1901564	Organonitrogen compound metabolic process	0.047	0.413968719	71
	GO:0050794	Regulation of cellular process	0.059	0.006725542	70
	GO:0044710	Single-organism metabolic process	0.044	0.729918588	70
	GO:1901360	Organic cyclic compound metabolic process	0.045	0.607606246	69
	GO:0046483	Heterocycle metabolic process	0.046	0.511664509	68
Cellular component	GO:0005623	Cell	0.047	0.262338243	159
	GO:0044464	Cell part	0.047	0.262338243	159
	GO:0005622	Intracellular	0.048	0.034069519	158
	GO:0044424	Intracellular part	0.048	0.05674525	157
	GO:0043226	Organelle	0.047	0.535722818	136
	GO:0043229	Intracellular organelle	0.047	0.535722818	136
	GO:0043231	Intracellular membrane-bounded organelle	0.048	0.400449801	128
	GO:0043227	Membrane-bounded organelle	0.047	0.404030601	128
	GO:0005737	Cytoplasm	0.046	0.749576798	120
	GO:0044446	Intracellular organelle part	0.049	0.303345435	98
	GO:0044422	Organelle part	0.048	0.322438964	98
	GO:0005634	Nucleus	0.057	0.00654983	86
	GO:0044444	Cytoplasmic part	0.041	0.981017574	84
	GO:0032991	Macromolecular complex	0.050	0.231134828	83
	GO:0043234	Protein complex	0.056	0.039327458	70
	GO:0044428	Nuclear part	0.057	0.050910536	52
	GO:0043228	Non-membrane-bounded organelle	0.050	0.290912385	52
	GO:0043232	Intracellular non-membrane-bounded organelle	0.050	0.290912385	52
	GO:0016020	Membrane	0.044	0.709496051	51
	GO:0031974	Membrane-enclosed lumen	0.048	0.471134956	44
Molecular function	GO:0003824	Catalytic activity	0.052	0.066198036	89
	GO:0005488	Binding	0.054	0.03289353	85
	GO:1901363	Heterocyclic compound binding	0.049	0.354708915	46
	GO:0097159	Organic cyclic compound binding	0.049	0.374406721	46
	GO:0016787	Hydrolase activity	0.061	0.022469004	44
	GO:0005515	Protein binding	0.066	0.008157207	41
	GO:0043167	Ion binding	0.069	0.006377994	37
	GO:0003676	Nucleic acid binding	0.046	0.582041834	31
	GO:0016740	Transferase activity	0.047	0.499203047	30
	GO:0043168	Anion binding	0.059	0.130714792	23
	GO:0036094	Small molecule binding	0.061	0.122019192	21
	GO:0017111	Nucleoside-triphosphatase activity	0.067	0.062770552	19
	GO:0016462	Pyrophosphatase activity	0.063	0.101576565	19
	GO:0016817	Hydrolase activity, acting on acid anhydrides	0.063	0.101576565	19
	GO:0016818	Hydrolase activity	0.063	0.101576565	19
	GO:0000166	Nucleotide binding	0.062	0.117341843	19
	GO:1901265	Nucleoside phosphate binding	0.062	0.117341843	19
	GO:0035639	Purine ribonucleoside triphosphate binding	0.069	0.057721559	18
	GO:0001883	Purine nucleoside binding	0.068	0.065096715	18
	GO:0032550	Purine ribonucleoside binding	0.068	0.065096715	18

At 96 h of TA-0410 infection of C88 leaf cells, the following upregulated differentially expressed genes were found to be enriched: those related to tryptophan metabolism, tyrosine metabolism, peroxisome, glycerophospholipid metabolism, purine metabolism, and other pathways. In filamentous fungi, the peroxisome was widely involved in melanin biosynthesis, cell wall integrity, fatty acid metabolism, and ROS homeostasis; these processes were closely related to fungal virulence (van der Klei and Veenhuis, 2013; Falter and Reumann, 2022; Figure 8B).

At 144 h of TA-0410 infection of C88 leaf cells, the main upregulated differentially expressed genes were mainly enriched in metabolic pathways, biosynthesis of secondary metabolites, ribosome, biosynthesis of amino acids, carbon metabolism, and other pathways. Fungal secondary metabolites were low molecular weight compounds produced by fungi that were toxic to plants. These metabolites show a high degree of diversity in terms of chemical structure, phytotoxic activity, and mode of toxic action. Metabolites of toxins and small-molecule virulence factors produced by pathogenic species were responsible for many of the devastating effects of plant diseases. New fungicides could be developed through the study of metabolite research (Bills and Gloer, 2016; Figure 8C).

For TB-1129-infected C88 leaf cells at 96 h, differentially expressed genes were mainly enriched in processes and pathways, such as metabolic pathways, biosynthesis of secondary metabolites, carbon metabolism, biosynthesis of amino acids, and protein processing in endoplasmic reticulum. At this stage, macromolecules, such as proteins, started to be synthesized to provide energy for the phloem germination (Figure 8D).

**FIGURE 9:**
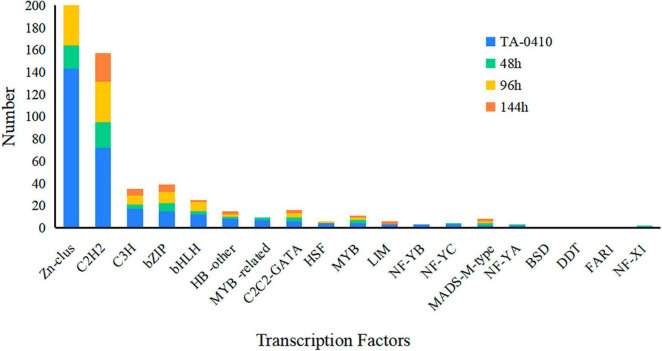
The number of transcription factors expressed at different time points in TA-0410.

TFs regulated different aspects of fungal development and virulence (John et al., 2021). We evaluated the differential expression of TFs at three time points after inoculation; the expression of *Zn-clus*, *C2H2*, *C3H*, *bZIP*, and *bHLH* was noticed. In fungi, the largest class of TFs was the zinc-coordinated “zinc finger,” which mainly controlled growth and development and was also essential for various stress responses and virulence (Xiong et al., 2015; John et al., 2021). *bZIP* was widely distributed in fungi and is one of the central regulators that played a role in a variety of biological processes in pathogenic fungi, such as fungal development, stress response, and pathogenicity (Gerin et al., 2021; Leiter et al., 2021). These TFs were found to be closely related to the development and pathogenicity of TA-0410 (Figure 9).

### 3.8 Proteomic analysis of TA-0410 infection in C88 leaf cells

**FIGURE 10:**
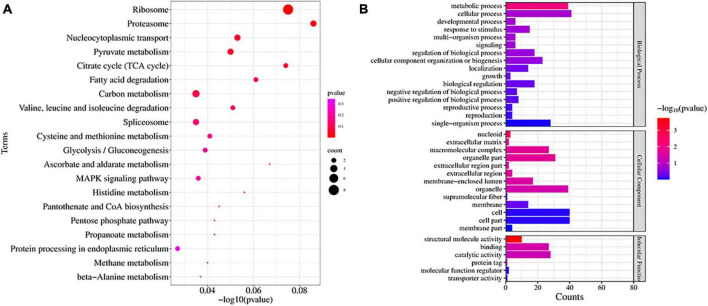
Proteins found enriched at 48 h after infection. **(A)** KEGG enrichment analysis. **(B)** GO enrichment analysis.

We studied the expression profile of proteins in C88 leaf cells infected with *A. solani* TA-0410 at 24 h and found enrichment of pathways, such as ribosome, proteasome, nucleocytoplasmic transport, and pyruvate metabolism (Figure 10A). The Biological Process was found to be enriched in cellular, metabolic, and single-organism processes, with the inclusion of cell, organelle, and membrane. The Molecular Function was mainly enriched in catalytic, structural and molecule activities, and molecular function regulator (Figure 10B). Among them, ribosomal protein S27a, in addition to playing a role in ribosome biogenesis and post-translational modification of proteins, was also involved in the ubiquitination pathway and had a C4-type zinc finger domain. The aspartate aminotransferase gene was demonstrated to be essential for the full pathogenicity of the oomycete pathogen *Phytophthora sojae* (Wang et al., 2016). Spermidine was critical for the growth, development, environmental adaptation, and virulence of *Fusarium graminearum* (Tang et al., 2021). Furthermore, it also affected the production and toxicity of aflatoxin in filamentous fungi (Majumdar et al., 2018). Nitronate monooxygenase, which contributed to the virulence, was also found to be induced in C88 leaf cells infected with TA-0410 strain. ClpA and ClpB played a very important role in the virulence of bacterial pathogens (Alam et al., 2021; Supplementary Table 5). Proteins expressed in early stages of the growth of TA-0410 were responsible for hyphae production and the germination of asexual conidia, whereas the expression of disease-related proteins prepared the pathogen for the pathogen to infect C88 leaf cells.

### 3.9 DEGs verified using qRT-PCR

**FIGURE 11:**
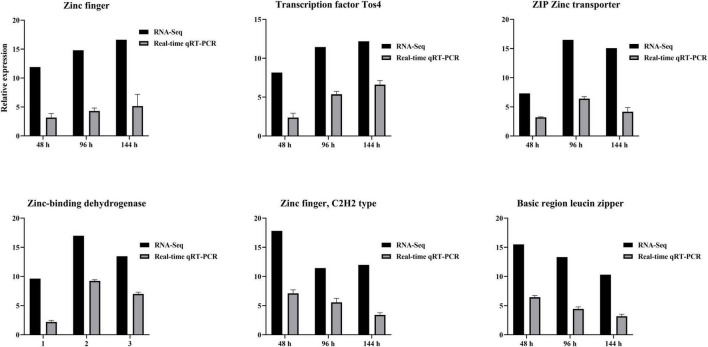
Verification of differential gene expression using qPCR at series times after infection with TA-0410.

RNA-seq results were verified using qRT-PCR. Zinc finger, Transcription factor Tos4, Zinc finger, C2H2 type, Basic region leucine zipper, ZIP Zinc transporter, and Zinc-binding dehydrogenase were expressed differentially at all three time points after TA-0410 infection of C88 leaf cells. The expression of Zinc finger and Transcription factor Tos4 increased and that of ZIP Zinc transporter and Zinc-binding dehydrogenase decreased with an increase in the infection time. The expression of malate synthase family and aldehyde dehydrogenase family proteins were the highest at 96 h. Overall, the qPCR results were consistent with those of RNA-seq results (Figure 11).

## 4 Discussion

*Alternaria* sp. is a genus of fungi with a global distribution that can cause blight in a variety of vegetables and crops, including potatoes, tomatoes, and tobacco, leading to significant agricultural losses. Although *A. solani* was the most recognized pathogen

causing early blight in potatoes and tomatoes, other species had also been identified on potato leaves showing symptoms of the disease, these included *A. alternata*, *Alternaria arborescens*, *Alternaria porri*, *Alternaria linariae*, and *Alternaria grandis* (Zheng et al., 2015; Landschoot et al., 2017; Edin et al., 2019). In this study, we discovered that both *A. solani* and *A. alternata* could be simultaneously isolated from typical diseased leaves of early blight in potatoes, up to 40% samples, indicating that the two types of fungi often occurred together under natural conditions, aggravating the occurrence of PEB. Pathogenicity assays and scanning electron microscopy observations revealed that *A. solani* had a stronger ability to infect potato leaves compared to *A. alternata*, these findings were consistent with previous reports (Stammler et al., 2014; Jindo et al., 2021). However, no other *Alternaria* species were found in our sample collection regions, this phenomenon indicated that the dominant species causing early blight disease in Yunnan in recent years were *A. solani* and *A. alternata*, but it could not be ruled out that other species had not been isolated due to host preference, sampling methods and other reasons. In our investigation, we also found that in some varieties with large planting areas, such as Li shu 6, Lishu 7, Qing shu 9, and C88, the disease of potato virus, late blight and purple top often occurred simultaneously with early blight disease, causing damage to the leaves. Therefore, it can be explained why *A. alternata* can be simultaneously isolated as a saprophyte on PEB lesions.

We assembled genome of *A. solani* and *A. alternata* via Illumina and PacBio HiFi sequencing methods. There were two *A. solani* genomes assembled into scaffold level in the GenBank database, ASM295215v1 and ASM283723v1, compared with the assembly results of TA-0410 in this study, ASM295215v1 has the largest N_50_ but no annotated data was available, and ASM283723v1 had the most CDS numbers but its N_50_ was too small. The genome assembled in this study (TA-0410) was the largest, with an N_50_ data close to ASM295215v1 and the number of annotated coding sequences (cds) close to ASM283723v1. Therefore, it was believed that the assembly effect of TA-0410 was better than that currently available in the database, and it could effectively carry out subsequent analysis work. By compare analyzing the genomes of *A. solani* TA-0410 and *A. alternata* TB-1129, we found that *A. solani* TA-0410 contained endemic genes related to conidial germination and pathogenicity, and these genes might be responsible for the ability of *A. solani* to infection.

*Alternaria* sp. infections in host organisms involved a series of complex and critical steps to cause disease. These included the stages of surface attachment, penetration through the cell wall, colonization within the host, and lesion formation. Some reports showed appressoria played a key role in the adsorption and penetration of fungi into host cells (Dean et al., 2012; Chethana et al., 2021a,b). During infection, the appressoria generated swelling pressures to provide the mechanical power for the pathogen to penetrate the outer epidermis and the cell wall of the host (Chethana et al., 2021a,b; Ryder et al., 2022), the pathogenicity mechanism of the appressoria produced by *A. solani* had rarely been reported. In this article, in the samples of 4 h after inoculation on the leaves, TA-0410 had formed obvious appressoria structures, while TB-1129 had not formed them even after 24 h of inoculation. Therefore, it was speculated that in most cases, TB-1129 could not complete effective infection due to external environmental stress or host resistance, while under conditions of wounds, the pathogen could successfully infect without forming appressoria, making it an effective conditionally pathogenic fungi for PEB. In addition, in further vitro experiments, we found that there was no antagonism between *A. solani* and *A. alternata* when they were co-cultured (data not shown in this study), so the co-infection of the two fungi could further expand and accelerate the production of PEB lesions.

The cell wall is located in the outermost layer of the plant and is mainly composed of polysaccharides such as hemicellulose, cellulose, and gliadin and a small amount of structural proteins, which is the first important line of defense against the invasion and expansion of pathogenic (Wan et al., 2021). Pathogens must secrete a series of cell wall-degrading enzymes to penetrate the cell wall and infiltrate the host, thereby accelerating their entry into the cells. In the genome of *A. solani* TA-0410, we identified a total of 68 cell wall-degrading enzymes, including pectinases, cutinases, and cellulases. Additionally, TA-0410 possesses 108 glycoside hydrolases, 4 polysaccharide lyases, and 3 carbohydrate esterases, among other enzymes linked to the breakdown of cell walls (Table 1 and Figure 6). Among them, glycoside hydrolase family, glycosyl hydrolase (cellulase C) family, and the glycosyl hydrolase (cellulase A) family were not present in *A. alternata* TB-1129, thus we hypothesized that this was another one of the reasons why *A. alternata* TB-1129 could not directly infect potatoes. The cell wall-degrading enzyme secreted by TA-0410 could degrade the cell wall and other tissues of the host plant, which was conducive to invasion, colonization and expansion. At present, much research progress had been made on the types of cell wall-degrading enzymes produced by many plant pathogenic (including fungi, oomycetes, and bacteria), the genes encoding them, and their pathogenic mechanisms. However, the importance of specific cell wall-degrading enzymes for pathogens varied markedly even among species of the same genus. Some cell wall-degrading enzymes have dual roles as both pathogenic factors and inducers of plant immunity, and resolving the structures of these proteins and identifying new classes will not only deepen the understanding of protein interactions in pathogenicity and host defense responses, but may identify new specific targets for plant protection and accelerate the development of new pharmacist for resistance to phytopathogenic (Kubicek et al., 2014).

In plant-microbe interaction, pathogens usually secreted a series of streptozotocins, effectors, growth regulators, and other active substances, which in turn directly or indirectly altered the normal physiological and biochemical responses of the host, causing host lesions (Meena et al., 2017), most probable due to induce PTI and ETI (Van der Nest et al., 2015; Witte et al., 2021), block certain key biosynthetic pathways (Guruceaga et al., 2019), ROS accumulation and transcription factors inhibition and activation, which were closely related to fungal virulence and are essential players in fungal infection of plants (Shelest, 2008; van der Klei and Veenhuis, 2013; Sadhu et al., 2019; John et al., 2021; Falter and Reumann, 2022). In our study, we revealed some pathways at different time points and 19 types transcription factors associated with *A. solani* TA-0410 conidial germination and virulence through a multi-omics analysis and verified six TE_S_ by qRT-PCR. These results can be further utilized in the analysis of genome remodeling and pathogenesis of the *A. solani*. Ubiquitin system had been also shown to play important roles in fungal development, stress resistance and fungal virulence (Chen et al., 2018; Yang et al., 2020; Cao and Xue, 2021). Thus, the ubiquitin-proteasome system related gene in *A. solani* found in this article may plays an important role in virulence. And further elucidation of its mechanism could be helpful for the development of pharmacological targets (Liu and Xue, 2011; Cao and Xue, 2021).

## 5 Conclusion

*Alternaria solani* as the primary pathogenic fungi causing PEB in Yunnan province in recent years. *A. alternata* was identified to be acting as a conditional pathogen, usually coexisting and infecting with *A. solani*, to accelerate infection. According to multi-omics analysis, cell wall-degrading enzymes, TFs, ubiquitination, and peroxidase genes, associated with conidia germination, appressorium formation, and virulence in *A. solani*, were mainly responsible for PEB occurrence. To sum, our results is conducive to a better understanding of the pathogen composition of PEB disease in Yunnan, enhanced comprehension of the pathogen infection characteristics and omics features of *A. solani* in potatoes, which could help in the development of fungicides and management practices.

## Data availability statement

The datasets presented in this study can be found in online repositories. The names of the repository/repositories and accession number(s) can be found below: https://www.ncbi.nlm.nih.gov/, OR486271, OR485642, JAMBQH000000000, JAHYXJ000000000, PRJNA1021048.

## Author contributions

QL: Writing – original draft, Writing – review & editing. YF: Data curation, Writing – original draft. JL: Data curation, Writing – review & editing. YH: Data curation, Investigation, Writing – review & editing. LS: Data curation, Investigation, Writing – review & editing. CT: Data curation, Validation, Writing – review & editing. JP: Data curation, Validation, Writing – review & editing. ZH: Methodology, Software, Writing – review & editing. ZL: Investigation, Resources, Writing – review & editing. CL: Funding acquisition, Writing – review & editing. DH: Funding acquisition, Writing – review & editing. WT: Funding acquisition, Writing – original draft, Writing – review & editing.

## Footnotes

1
http://www.cazy.org
